# Dynamic features of human mitochondrial DNA maintenance and transcription

**DOI:** 10.3389/fcell.2022.984245

**Published:** 2022-09-07

**Authors:** Mansour Akbari, Hilde Loge Nilsen, Nicola Pietro Montaldo

**Affiliations:** ^1^ Department of Medical Biology, Faculty of Health Sciences, UiT-The Arctic University of Norway, Tromsø, Norway; ^2^ Department of Clinical Molecular Biology, Institute of Clinical Medicine, University of Oslo, Oslo, Norway; ^3^ Unit for precision medicine, Akershus University Hospital, Nordbyhagen, Norway; ^4^ Department of Microbiology, Oslo University Hospital, Oslo, Norway

**Keywords:** mitochondria, DNA repair, base excision repair (BER), base excision repair (BER)glycosylases, transcription, mitochdrial damage

## Abstract

Mitochondria are the primary sites for cellular energy production and are required for many essential cellular processes. Mitochondrial DNA (mtDNA) is a 16.6 kb circular DNA molecule that encodes only 13 gene products of the approximately 90 different proteins of the respiratory chain complexes and an estimated 1,200 mitochondrial proteins. MtDNA is, however, crucial for organismal development, normal function, and survival. MtDNA maintenance requires mitochondrially targeted nuclear DNA repair enzymes, a mtDNA replisome that is unique to mitochondria, and systems that control mitochondrial morphology and quality control. Here, we provide an overview of the current literature on mtDNA repair and transcription machineries and discuss how dynamic functional interactions between the components of these systems regulate mtDNA maintenance and transcription. A profound understanding of the molecular mechanisms that control mtDNA maintenance and transcription is important as loss of mtDNA integrity is implicated in normal process of aging, inflammation, and the etiology and pathogenesis of a number of diseases.

## 1 Introduction

**FIGURE 1:**
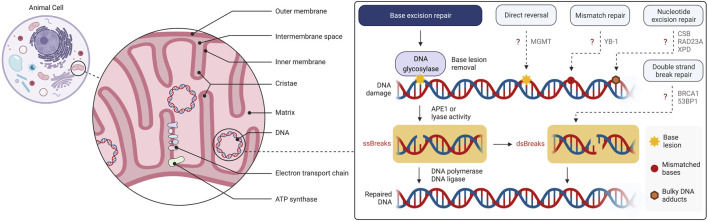
Overview of the mitochondrial structure and mtDNA damage and repair. Base excision repair (BER) is the primary mtDNA repair pathway. Canonical forms of the nuclear DNA repair pathways, mismatch repair (MMR), nucleotide excision repair (NER), direct reversal (DR) and double strand break repair (DSBR), have not been demonstrated to operate in mitochondria although individual enzymes of these pathways may be present. The figure has been generated using Biorender.com.

Research on mitochondria and their gene architecture has led to the hypothesis that this organelle has evolved from an endosymbiotic event 1.5 billion years ago, when an archaea-like host engulfed a proteobacterium-like ancestor (Lang et al., 1999; Dyall et al., 2004). Thus, mitochondria have their own genome and mitochondrial respiratory chain complexes have high homology with those found in bacteria. Further, to some extent mitochondria rely on their own ribosomes, tRNAs and associated protein factors as protein translation machinery resembling the bacterial ancestors. Mitochondria have an outer and an inner membrane which separate the innermost compartment, the mitochondrial matrix, from the cytosol (Figure 1). Ions and small uncharged molecules can freely pass through the outer membrane through porins, the pore-forming outer membrane proteins. Larger molecules and proteins, require specific translocases for import. The inner membrane is a tight and selective barrier that forms extensive invaginations into the matrix called cristae (Figure 1). Most complexes of the electron transport chain (ETC) are embedded into or are tightly associated with the inner membrane ETC complexes establish the electrochemical gradient across the inner membrane that is then utilized by the ATP synthase to generate ATP, the “energy currency” of the cells. This process is called oxidative phosphorylation (OXPHOS).

Mitochondria also play a central role in several other key biological processes such as regulation of apoptosis (Gahl et al., 2016), cytoplasmic calcium buffering (Drago et al., 2012), and reactive oxygen species (ROS) mediated signaling pathways (Shadel and Horvath, 2015). The intracellular localization, biogenesis, and bioenergetics of mitochondria are largely determined through interplay between these processes and are tightly connected to mitochondrial function and cellular need for energy (Fernandez-Vizarra et al., 2011).

Our genome resides in the nucleus and in the mitochondria. The mitochondrial genome is a 16.6 kb, maternally inherited, intron-less, circular DNA. Mitochondrial DNA (mtDNA) is packaged with mitochondrial transcription factor A (TFAM) and other proteins into structures called nucleoids tightly associated with the inner mitochondrial membrane (Kukat et al., 2011).

Most of the mtDNA has been transferred to the nuclear genome during the evolution (Lopez et al., 1994; Mourier et al., 2001). Mitochondria contain approximately 1,200 proteins (Rath et al., 2021); nearly all of them are encoded by nuclear DNA and transported into the mitochondria. Human mtDNA has retained 13 protein coding genes encoding subunits of electron transport chain complexes CI, CIII, CIV, and CV or ATP synthase, which are all essential for OXPHOS (Anderson et al., 1981). Each mitochondrion contains multiple copies of mtDNA. The mtDNA copy number varies significantly among different tissues (D'Erchia et al., 2015). Altered mtDNA copy number (Grady et al., 2018; Filograna et al., 2021) and mtDNA depletions caused by disturbed mitochondrial dNTP pool (Viscomi and Zeviani, 2017) are associated with human disease. Many pathogenic mtDNA mutations have been reported (MITO-MAP). Single nucleotide mutations (Goto et al., 1990) and large scale deletions (Moraes et al., 1989) can cause diseases with a wide variety of clinical manifestations ranging from myopathy to neurological disorders (Gorman et al., 2016). MtDNA mutations can be present in a fraction of total mtDNA or in all mtDNA molecules referred to as mtDNA heteroplasmy and homoplasmy, respectively.

Accumulation of various forms of mtDNA damage correlates with aging (DeBalsi et al., 2017), indicating that diminished mtDNA integrity is tightly connected to normal process of tissue aging. Replication errors followed by clonal expansion of mutated mtDNA driven by positive selection and random genetic drift, are commonly thought prominent mechanisms driving the accumulation of mtDNA mutation in various tissues with age (Elson et al., 2001; Ameur et al., 2011; DeBalsi et al., 2017).

The importance of mtDNA for normal cellular and organismal function is illustrated by the observation that defect in the core components of the mtDNA replication and repair machineries cause diseases with a range of clinical presentations from severe infantile multi-tissue diseases to adult-onset progressive degeneration of muscles and neurons (Takashima et al., 2002; Das et al., 2010; Gao et al., 2011; Sykora et al., 2011; DeBalsi et al., 2017; Viscomi and Zeviani, 2017).

The maintenance of mtDNA involves mechanisms distinct from nuclear DNA repair and maintenance systems. Mitochondria lack the full repertoire of DNA repair pathways present in the nucleus, and mtDNA homeostasis involves processes that control mitochondrial morphology and dynamics (Sabouny and Shutt, 2021).

## 2 Mitochondrial maintenance: Mitochondrial dynamics, clearance, and repair

### 2.1 Mitochondrial DNA repair

In the nuclear genome, lesions are repaired by different DNA repair pathways depending on the nature of the lesion, phases of the cell cycle, and posttranslational modifications of the repair proteins (Akbari et al., 2009; Mjelle et al., 2015; Carter and Parsons, 2016; Fowler et al., 2022). The main pathways include mismatch repair (MMR), nucleotide excision repair (NER), base excision repair (BER) and the double strand break (DSB) repair (DSBR) pathways homologous recombination (HR) or non-homologous end joining (NHEJ). Some enzymes involved in these nuclear pathways have been reported to localize in mitochondria. It is not known if the presence of these enzymes represents non-canonical forms of the nuclear pathways or different mechanisms which might be exclusive to mitochondria. For instance, low-level repair activity on nicked heteroduplex substrate with a GT or a GG mismatch was identified in rat liver mitochondrial lysate. Similar to the nuclear MMR, the activity was mismatch-selective, bi-directional, ATP dependent and EDTA-sensitive (Mason et al., 2003). However, later it was found that this MMR activity was dependent on the Y-box binding protein (YB-1) as its depletion led to a decrease of MMR correction activity in the mitochondrial extracts and an increase in mtDNA mutations (de Souza-Pinto et al., 2009), thus suggesting an atypical mechanism.

Some enzymes acting in nuclear NER have been reported to localize in the mitochondria. Cockayne syndrome group B (CSB) localizes in the mitochondria, where it stimulates mtDNA stability likely by facilitating BER (Aamann et al., 2010). RAD23 homolog A (RAD23A) can be recruited to mitochondria following induction of oxidative mtDNA damage and upon mt-Ox treatment. Accordingly, *Rad23a* ^*−/−*^ mouse embryonic fibroblasts (MEFs) showed elevated 8-oxoG levels in mtDNA compared to *Rad23a* ^*+/+*^ cells (Wisnovsky et al., 2016). Xeroderma pigmentosus group D (XPD) localizes in mitochondria and protects the mitochondrial genome from oxidative DNA damage (Liu et al., 2015). Thus, a small selection of NER proteins appears to be present in the mitochondria, but several enzymes and activities required for NER are lacking.

A DNA end-joining activity for repair of DSBs have been reported in mitochondrial extracts from mammalian cells (Lakshmipathy and Campbell, 1999) and Breast cancer type 1 susceptibility protein (BRCA1) and p53-binding protein 1 (53BP1) have been reported to be in the mammalian mitochondria (Coene et al., 2005; Youn et al., 2017).

Some biochemical evidence suggests O^6^-methylguanine (O^6^-MeG) lesions removal in mitochondria (Myers et al., 1988). However, O6-methylguanine DNA methyltransferase (MGMT), the enzyme responsible for O^6^MeG direct reversal (DR) in the nucleus, has not been verified in the mitochondrial compartment, and the existence of DR in mammalian mitochondria remains uncertain (Satoh et al., 1988; LeDoux et al., 1992). In conclusion, the presence of NER, MMR, and DSBR in mitochondria and how mitochondrial deal with lesions typically removed by these pathways need further investigation. There is, however, substantial evidence for the presence of BER in mitochondria (Akbari et al., 2008; Kazak et al., 2012).

BER is the key pathway for repair of a number of different types of DNA base lesions, as well as apurinic-apyrimidinic sites (AP sites), and single-strand DNA breaks (Krokan and Bjoras, 2013) (Figure 1). BER is initiated by removal of damaged base by a DNA glycosylase. After removal of the substrate base, apurinic endonuclease 1 (APE1) incises immediately 5′ to the AP site leaving a gap in DNA that is filled by DNA polymerase. The gap filling can be in the form of single nucleotide insertion, or through incorporation of several nucleotides called long-patch BER (Akbari et al., 2008). The final step in BER is the end-joining or ligation of the DNA phosphodiester backbone by DNA ligase (Krokan and Bjoras, 2013).

Mechanistically, nuclear and mitochondrial BER are identical, and they share most of their proteins. However, mitochondrial BER seems to be somewhat less robust than the nuclear BER (Yakes and Van Houten, 1997; Akbari et al., 2014; Akbari et al., 2015).

In an early study, the repair capacity for lesions generated by treatment with 200 μM hydrogen peroxide on mtDNA was investigated (Yakes and Van Houten, 1997). Following the treatment, a 16.2 kb mtDNA fragment and a 17.7 kb fragment flanking the β-globin gene were PCR amplified and the amplicons were used to measure the level of damage and repair in the target DNA regions. When the treatments were limited to 15 min, mtDNA damage was repaired with similar kinetics as the nuclear fragment. However, following a 60 min treatment, damage to the nuclear fragment was completely repaired within 90 min, whereas no DNA repair in the mitochondrion was detected (Yakes and Van Houten, 1997), suggesting that prolonged DNA damage may exhaust the mitochondrial BER capacity.

In another study, mitochondrial BER capacity was investigated using a circular DNA substrate containing uracil at a specific position in highly purified mitochondrial extracts from human U2OS and HeLa cell lines (Akbari et al., 2014). DNA ligation was significantly slower than the preceding mitochondrial BER steps. Moreover, overexpression of DNA ligase III in mitochondria improved the rate of overall BER, and increased cell survival after extraneously induced oxidative stress (Akbari et al., 2014). An efficient ligation step in BER is particularly important to prevent the accumulation of nicks in mtDNA that can give rise to DSBs and block DNA replication and transcription.

Mammalian cells contain at least 11 different DNA glycosylases. Nuclear and mitochondrial isoforms of DNA glycosylases are encoded by the same gene; however, mitochondrial DNA glycosylases are usually generated by alternative splicing and alternative transcription initiation (Nilsen et al., 1997; Takao et al., 1999; Ohtsubo et al., 2000). Mitochondrial DNA glycosylases include DNA glycosylases 8-oxoguanin DNA glycosylase (OGG1), MutY homologue (MYH), Endonuclease III-like protein 1 (NTHL1), Endonuclease VIII-like protein 1 (NEIL1), Endonuclease VIII-like protein 2 (NEIL2), that initiate the repair of oxidative DNA base lesions; uracil-DNA glycosylase 1 (UNG1) that initiate the repair of uracil; Alkyladenine DNA glycosylase (AAG) which repairs alkylated DNA lesions.

APE1 is the prominent endonuclease responsible for the processing of the AP sites generated following the removal of damaged base by DNA glycosylase. APE1 contains a nuclear localization signal within its N-terminal domain that directs the protein within the nucleus (Jackson et al., 2005). APE1 is imported into mitochondria through the translocase of the outer membrane (TOM) pore complex (Li et al., 2010). APE1 may contain a putative mitochondrial localization signal within amino acids 289–318 in its C- terminus (Li et al., 2010).

Abortive DNA-ligase activity during replication and BER could result in 5′-AMP–DNA overhang. Aprataxin (APTX), a member of the histidine triad superfamily, removes 5′-AMP from DNA. Defect in APTX causes the ataxia oculomotor apraxia (AOA1) an autosomal recessive spinocerebellar ataxia syndrome (Date et al., 2001). APTX is present in both the nucleus and mitochondria (Sykora et al., 2011). Biochemical experiments suggest that contrary to nuclear DNA repair where long-patch BER can remove 5′-AMP from DNA as part of the displaced 5′ strand (5′flap), mitochondrial BER is not able to compensate for APTX deficiency resulting in the persistent 5′-AMP lesions in mtDNA and may thus contribute to AOA1 pathology (Akbari et al., 2015).

Polymerase gamma (Pol γ) is a Family A replicative DNA polymerase and functions in mtDNA replication and repair. Pol γ is a heterotrimer composed of a 140 kDa catalytic subunit and a homodimer 110 kDa accessory subunit (POLG2) that increases processivity of DNA synthesis (Krasich and Copeland, 2017). Pol γ contains a C-terminus polymerase domain and an N-terminus proofreading 3′–5′ exonuclease domain. The catalytic subunit has an associated lyase activity that catalyzes the removal of 5′-deoxyribose phosphate residue from incised AP site during BER (Longley et al., 1998).

DNA polymerases other than Pol γ might exist in mitochondria in specific tissues or transiently in response to stress (Krasich and Copeland, 2017; Sykora et al., 2017; Zheng et al., 2020). Functional Pol γ is, however, indispensable for normal mitochondrial and organismal function indicating a prominent role of Pol γ in mtDNA maintenance and integrity (DeBalsi et al., 2017).

In higher eukaryotes, there are three distinct DNA ligases: DNA ligase I is required for the ligation of Okazaki fragments during lagging strand DNA synthesis and is involved in several DNA repair pathways; DNA ligase III has been associated with BER and NER; DNA ligase IV is mainly involved in NHEJ. A mitochondrial isoform of DNA ligase III is responsible for DNA ligation during mtDNA replication and repair (Lakshmipathy and Campbell, 2001; Gao et al., 2011; Simsek et al., 2011).

### 2.2 Disconnection of mtDNA damage and mtDNA mutation load

In nuclear DNA, unrepaired lesions can be fixed as mutations following DNA replication and somewhat by the process of DNA repair itself. In mtDNA this link between DNA lesion and the generation of mutation appears to be weak despite less robust DNA repair activity in mitochondria compared with the nucleus (Itsara et al., 2014; Valente et al., 2016; Kauppila et al., 2018).

Mitochondrial dysfunction is a pathological hallmark of several neurodegenerative diseases and is often associated with apoptosis and neuronal loss. The effect of mtDNA instability in mitochondrial dysfunction and neurodegeneration was investigated in mice engineered to generate high levels of AP sites in mtDNA in forebrain neurons by tissue-specific expression of a mutated version of the mitochondrial uracil-DNA glycosylase that removes thymine instead of uracil from mtDNA (Lauritzen et al., 2010). High levels of AP sites in mtDNA from the transgenic mice was confirmed by a probe that reacts with the exposed aldehyde group at AP sites. These mice showed aberrant mitochondrial function, neuronal death, and behavioral deficits consistent with the crucial role of mitochondria in neuronal function and survival. MtDNA mutation load was tested by PCR amplification of cytochrome B gene followed by cloning, and sequencing of the amplicons. The results failed to detect any differences in the mutation frequency between mtDNA from transgenic mice compared with the wild-type littermates (Lauritzen et al., 2010). The authors speculated that the lack of mutation elevation could be due to efficient repair of AP sites, though this conclusion was not supported by mtDNA copy number analysis to rule out degradation of damaged mtDNA.

The inner mitochondrial membrane is commonly thought to be the primary site of ROS production. ROS can generate a plethora of pre-mutagenic DNA lesions (Akbari and Krokan, 2008; Murphy, 2009), including 8-oxodG, a common oxidative DNA base lesion which can mispair with adenine during DNA replication giving rise to G to T transversion mutations (Bjelland and Seeberg, 2003). Consistent with the relatively high number of oxidative base damage events in mtDNA, and the central function of OGG1 in 8-oxodG repair, mtDNA from OGG1-deficient mice contained 20-fold more 8-oxodG than mtDNA from wild-type animals (de Souza-Pinto et al., 2001).

DNA glycosylases OGG1 and MYH functionally cooperate to prevent G to T mutations arising from 8-oxoG lesions. Mice deficient in both MYH and OGG1 DNA glycosylases display strong tumorigenesis and severely reduced lifespan (Xie et al., 2004). Most of the tumors showed G to T transversion mutations in nuclear DNA determined by PCR amplification of the nuclear *k-RAS* gene followed by nucleotide sequencing analysis of cloned PCR products (Xie et al., 2004). A separate study, using PCR-based methods, failed to detect elevated mtDNA mutations in *myh* and *ogg1* double knockout mice. However, biochemical analysis of the mitochondrial ETC complex I activity and mitochondrial respiration measurements demonstrated a significant reduction in respiratory capacity in brain mitochondria from the adult double knockout mice (Halsne et al., 2012).

In a recent study, CRISPR-Cas9 based gene editing was used to generate cells lacking DNA glycosylase OGG1 that removes 8-oxoG from DNA, or MTH1 that hydrolyses 8-oxo-dGTP to 8-oxo-dGMP to prevent its insertion into DNA. *In vivo* mitochondrial BER activity was measured in OGG1-and MTH1-deficient cells using a cell-and mitochondrial-permeable fluorescent probe that reacts with AP sites in mtDNA (Jun et al., 2022). Following exposure of the cells to an exogenous oxidating compound, mitochondrial BER was greatly elevated in the MTH1 KO cell line demonstrating the importance of sanitizing 8-oxoG precursors in mitochondria. The OGG1 KO cells showed lower BER activity than both MTH1 KO cells and the wild-type control cells resulting in the accumulation of 8-oxoG lesions in mtDNA (Jun et al., 2022).

Thus, despite recent reports showing that BER deficiency does not lead to increased mutation load in mtDNA or mtRNA in mice (Kauppila et al., 2018), the fact that BER, as well as the nucleotide pool sanitizing enzymes MTH1 and deoxyuridine nucleotidohydrolase (dUTPase) that eliminates dUTP, exist in mitochondria indicate the importance of preventing and repairing base lesions in mtDNA (Kang et al., 1995; Ladner and Caradonna, 1997; Ichikawa et al., 2008; Jun et al., 2022).

**FIGURE 2:**
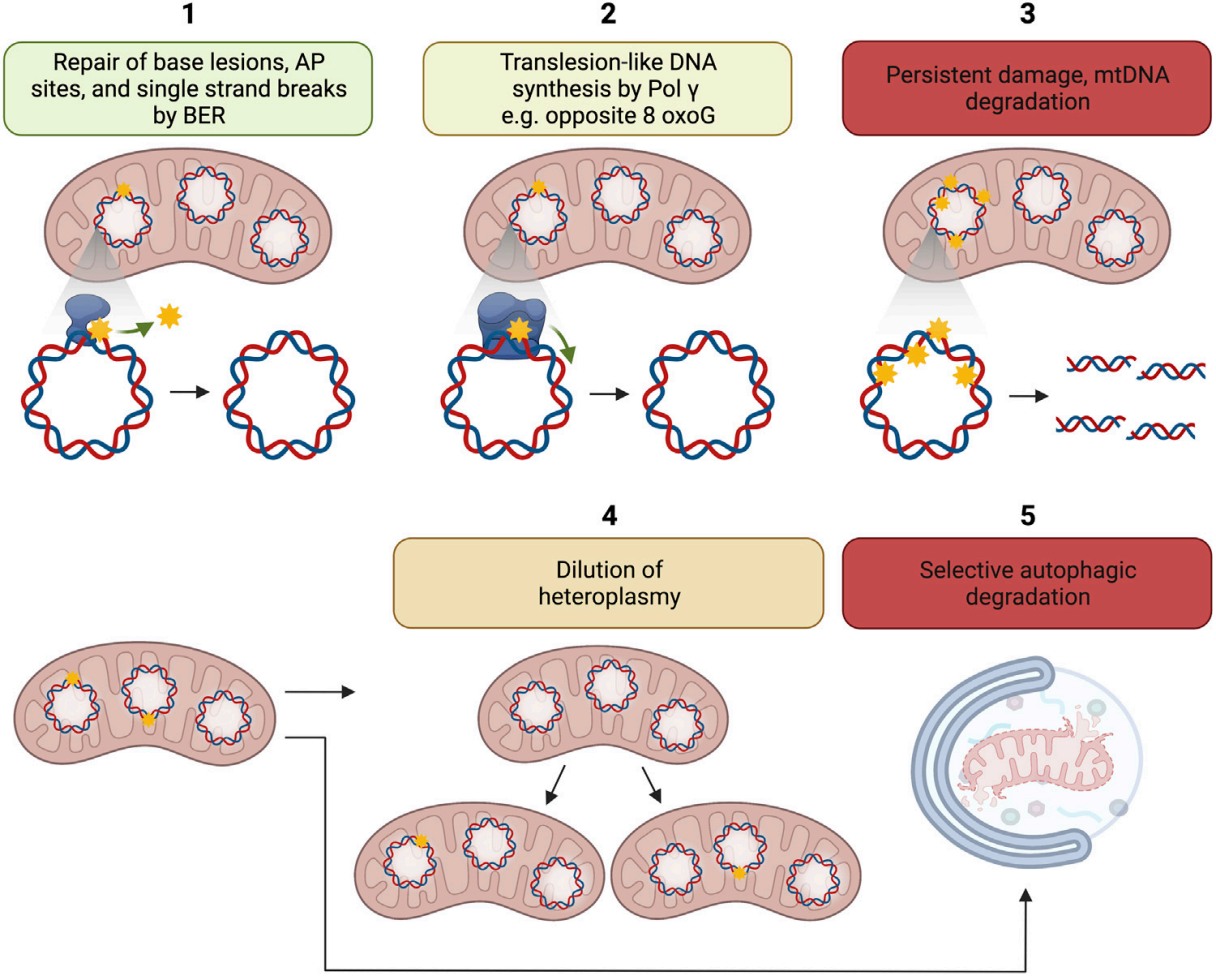
Putative mechanisms for disconnection between mtDNA damage and mtDNA mutation load. 1. Repair of base lesions, AP sites, and single strand breaks by BER; 2. Translesion like DNA synthesis by Pol γ e.g., opposite 8-oxoG; 3. Persistent damage may lead to the degradation of damaged mtDNA; 4. Dilution of heteroplasmy, i.e., ratio of mutant to wild-type mtDNA by mitochondrial dynamics; 5. Possible selective autophagic degradation of mitochondria with high levels of mtDNA damage. The figure has been generated using Biorender. com.

Several mechanisms may account for the disconnection between mtDNA damage and mutation (Figure 2). Pol γ is a high fidelity DNA polymerase showing an error rate of ∼3 × 10^–5^ (Johnson and Johnson, 2001) comparable with the replicative DNA polymerases Polε and Polδ each displaying an error rate of 10^−5^–10^–6^ and 10^−4^–10^–6^, respectively (Burgers and Kunkel, 2017). Because 8-oxoG can mispair with adenine during DNA synthesis and 8-oxoG is highly relevant mtDNA lesion, biochemical experiments have been caried out to understand the efficiency and fidelity of 8-oxoG bypass. Pol γ has been found to display moderate DNA translesion-like synthesis activity and is able to insert the correct dCMP (≈ dAMP ≫ dGMP) opposite 8-oxo-dG, thus counteracting the pre-mutagenic capacity of 8-oxoG in mtDNA (Figure 2). No dTMP incorporation was observed (Graziewicz et al., 2007).

MtDNA degradation is another potential mechanism disconnecting mtDNA damage to mtDNA mutation accumulation (Figure 2).

It has long been known that mtDNA has a higher turnover than nuclear DNA (Gross et al., 1969). In an early study, high-performance liquid chromatography was used to measure the amount of 8-oxoG in mtDNA isolated from rat liver mitochondria. The results showed that long and intact circular mtDNA contained much lower amounts of 8-oxoG than shorter fragmented mtDNA molecules (Suter and Richter, 1999) suggesting elevated turnover of highly damaged mtDNA molecules. Evidence now supports the idea that degradation of highly damaged mtDNA is an important mechanism for the preservation of mtDNA integrity (Shokolenko et al., 2009; Shokolenko et al., 2013).

Several mechanisms have been shown to facilitate the degradation of damaged mtDNA. DNA double-strand breaks (DSBs) occur in mtDNA because of defective replication as well as through ROS generated DNA damage. Mitochondria lack the robust DSB repair that is present in the nucleus. Generation of target specific mtDNA DSBs resulted in the loss of the damaged mtDNA molecules because of extensive mtDNA degradation (Moretton et al., 2017). Components of the mtDNA replisome i.e., Pol γ, DNA helicase TWINKLE, and the 5′-3′exonuclease MGME1, all seem to contribute to the degradation of linear mtDNA generated following DSBs (Nissanka et al., 2018; Peeva et al., 2018).

TFAM is an abundant mitochondrial protein that coats the entire mtDNA (Kukat et al., 2011), and regulates mtDNA copy number (Ekstrand et al., 2004). TFAM may promote the degradation of mtDNA containing AP sites. Purified TFAM complexed with AP site containing oligodeoxynucleotides, reduced the half-life of AP sites by DNA strand incision at AP sites. Chemical trapping of the reaction by sodium cyanoborohydride suggested that the AP site incision reaction occurred via Schiff base intermediate which is a necessary step in the β-elimination cleavage by AP lyases (Xu et al., 2019). Considering the central role of TFAM in mtDNA metabolism and the emerging importance of mtDNA degradation as an active mechanism to counteract mtDNA damage accumulation, more research is warranted to determine the biological significance of this finding.

### 2.3 Mitochondrial dynamics in mtDNA maintenance

Mitochondria fuse together to form larger mitochondria and build tubular networks. They also undergo fission to form smaller mitochondria. These events, together with the mechanisms that control mitochondrial quality are collectively referred to as mitochondrial dynamics and are controlled by a number of intra- and extracellular cues (Giacomello et al., 2020). Defect in the regulators of mitochondrial dynamics cause disease, often in the form of severe neuromuscular and neurodegenerative disorders (Dorn and Dang, 2022; Romanello and Sandri, 2022).

Mitochondrial fusion and fission involve a group of dynamin-like proteins and GTPases. The key proteins in fusion include the outer mitochondrial membrane proteins mitofusin 1 (MFN1) and mitofusin 2 (MFN2), and the inner mitochondrial membrane protein optic atrophy type 1 (OPA1) (Koshiba et al., 2004). The cytosolic dynamin-related protein 1 (DRP1), and several mitochondrial outer membrane DRP1-binding proteins or receptors are key regulators of mitochondrial fission (Loson et al., 2013).

Mitochondrial dynamics exert a profound effect on mtDNA homeostasis manifested by mtDNA copy number and integrity. Mitochondrial dynamics also controls the distribution of normal and mutant mtDNA in the cellular mitochondrial population, i.e., heteroplasmy, to prevent that the number of mutant mtDNA molecules in each mitochondrion exceed a threshold that could impair mitochondrial function (Figure 2 box 4) (Amati-Bonneau et al., 2008; Hudson et al., 2008; Chen et al., 2010; Yu-Wai-Man et al., 2010; Elachouri et al., 2011; Peralta et al., 2018; Sabouny and Shutt, 2021; Sidarala et al., 2022).

Mutations in *OPA1* cause dominant optic atrophy a disease affecting retinal ganglion cells. Clinical presentation of the disease includes optic atrophy, progressive external ophthalmoplegia, ataxia, deafness, and sensory-motor neuropathy (Alexander et al., 2000; Delettre et al., 2000). Mitochondria in skeletal muscle from patients with OPA1 mutation show mosaic cytochrome c oxidase deficiency and contain mtDNA with multiple deletions (Amati-Bonneau et al., 2008; Hudson et al., 2008). Fibroblasts from patients with OPA1 mutation display mitochondrial network fragmentation (Bonifert et al., 2014; Liao et al., 2017).

Homozygous *Opa1* mutant mice die *in utero* during embryogenesis, but heterozygous *Opa1* mutants display the main features of human dominant optic atrophy including abnormal mitochondrial morphology, disorganized cristae structure, mitochondrial dysfunction and mtDNA instability (Alavi et al., 2007; Davies et al., 2007; Chen et al., 2012).

Complete depletion of MFN1 and MFN2 in mice cause embryonic lethality (Chen et al., 2003). Target specific deletion of *Mfn1* and *Mfn2* in skeletal muscle during development resulted in severe mtDNA depletion in muscle that preceded physiological abnormalities (Chen et al., 2010). In another study, β-cells specific deletion of *Mfn1* and *Mfn2* in mice showed that MFN1 and MFN2 control glucose homeostasis in a manner that was connected to their role in the preservation of mtDNA number by regulating the expression of *Tfam* (Sidarala et al., 2022). Thus, MFN1 and MFN2 appear to control mitochondrial homeostasis through regulation of mitochondrial structure as well as mtDNA copy number maintenance.

FBXL4 is a member of F-box protein family. Mutations in *FBXL4* were identified in patients with lactic acidosis and severe encephalopathic syndrome (Bonnen et al., 2013; Gai et al., 2013). Analysis of patient tissue samples identified various mitochondrial abnormalities including reduced mtDNA content and impaired mitochondrial morphology (Bonnen et al., 2013; Gai et al., 2013; Antoun et al., 2016). However, it is possible that mitochondrial phenotypes in FBXL4 deficient cells might be related to a putative role of FBXL4 in controlling mitochondrial turnover by autophagy rather than a direct role of FBXL4 in mitochondrial biogenesis and mtDNA copy number regulation (Alsina et al., 2020).

### 2.4 Mitochondrial clearance

Dysfunctional or superfluous mitochondria can be degraded via bulk autophagy or mitochondria-specific autophagy (mitophagy) (Figure 2 box 5) (Li et al., 2022). Mutations in mitophagy regulators, such as PTEN-induced putative kinase 1 (PINK1) and the cytosolic E3 ligase Parkin, are known as causal genes for familiar Parkinson disease (Kitada et al., 1998; Valente et al., 2004; Narendra et al., 2010). Mitochondria harboring mtDNA damage seem to be able to escape mitophagy and propagate in the cell. The missed clearance of these mutation-bearing mitochondria causes somatically mutated mtDNA to undergo clonal expansion during aging and thereby cause mosaic respiratory chain deficiency in various tissues (Larsson, 2010). Patients with inherited mtDNA depletion syndrome suffer from strong tissue-specific reductions in mtDNA levels (Viscomi and Zeviani, 2017). Thus, whether, and to what extent, mtDNA damage could activate mitophagy needs further investigation.

### 2.5 MtDNA maintenance in disease

Mitochondrial DNA maintenance defects (MDMDs) are a class of diseases caused by pathogenic mutations in nuclear-encoded mtDNA maintenance proteins. These diseases are characterized by substantial reduction in mtDNA copy number, accumulation of mutations, and large-scale deletions. MDMDs can be autosomal recessive or dominantly inherited, and they can cause a wide range of symptoms. MDMDs have been related to pathogenic variants in 20 nuclear genes that are important for mtDNA maintenance. These mutations have been found in three gene categories: genes for mtDNA replication machinery enzymes (POLG, POLG2, TWNK, TFAM, RNASEH1, MGME1, and DNA2); genes encoding proteins involved in mitochondrial nucleotide pool synthesis (TK2, DGUOK, SUCLG1, SUCLA2, ABAT, RRM2B TYMP, SLC25A4, AGK, MPV17); and genes encoding proteins involved in mitochondrial dynamics (OPA1, MFN2, FBXL4) (reviewed in (El-Hattab et al., 2017)).

New evidence suggests that BER causes accumulation of single-stranded DNA breaks in an age-dependent manner and contributes to Parkinson disease–like pathology in a *C. elegans* model. NTH-1 DNA glycosylase mediated mitochondrial and nuclear genomic instability promoting degeneration of dopaminergic neurons in old nematodes. Conversely, NTH-1 deficiency protects against α-synuclein-induced neurotoxicity, maintaining neuronal function with age (SenGupta et al., 2021). In humans, whole-exome sequencing analysis from two independent cohorts of clinically validated idiopathic PD and controls, the Norwegian ParkWest cohort (*n* = 411) and the North American Parkinson’s Progression Markers Initiative (*n* = 640) showed that, compared to controls, individuals with idiopathic PD had a significant polygenic enrichment of rare, nonsynonymous variants in the genes encoding enzymes involved in mtDNA maintenance (Gaare et al., 2018). This work suggests that the results found in *C. elegans* might be relevant in human settings. However, more studies in human cells are required to confirm such putative mechanisms.

### 2.6 Methods for measuring mtDNA topology, damage, and repair

To interpret the functional implications of mtDNA maintenance mechanisms and their relevance to disease, it is important to be aware of both deliverables and limitations of the various assays used to detect mtDNA topology and damage. Here we review the assays used for measuring mtDNA topology, copy number. and mtDNA damage.

### 2.6.1 Assays for measuring mtDNA topology and copy number

In 1963, transmission electron microscopy studies of chicken embryo mitochondria revealed that mitochondria have their own DNA (Nass and Nass, 1963a; Nass and Nass, 1963b). Transmission electron microscopy in combination with other techniques and enzymatic assays has been used in mtDNA topology and replication studies (Kasamatsu et al., 1971; Kosar et al., 2021).

Southern blot is a technique where DNA fragments of different lengths are separated by size via gel electrophoresis, transferred to a membrane and visualized by hybridization with radioactive or non-radioactive labeled sequence-specific DNA probes. One dimensional agarose gel (1D gel) electrophoresis technique allows separation of molecules roughly according to their mass. 1D gel electrophoresis of mtDNA provides a qualitative distribution of total mtDNA topoisomers, catenated circles, nicked circular mtDNAs, linear and supercoiled circular DNA molecules (Pohjoismaki et al., 2006). Gel electrophoresis of mtDNA linearized with restriction enzymes provides information about mtDNA content (Wheeler et al., 2019) and large deletions (Kaukonen et al., 2000).

Initiation, elongation, and termination of DNA replication are each associated with distinct, nonlinear DNA structures. The two-dimensional agarose gel (2D gel) electrophoresis technique, developed by Brewer and Fangman in 1987 (Brewer and Fangman, 1987) allows separation of these nonlinear structures based on two dimensions: The first dimension gel is run at low voltage in low percentage agarose (0.4%) to separate DNA molecules in proportion to their mass; the second dimension is run at high voltage in a higher agarose concentration (≥1%) gel in the presence of ethidium bromide (or a similar intercalating agent), so that the mobility of a non-linear molecule is greatly influenced by its shape (Brewer and Fangman, 1987). This technique allows the resolution of nonlinear replication intermediates in specific and predictable ways and was adapted to study mtDNA replication mechanisms (Han and Stachow, 1994; Holt et al., 2000). The simplest type of replication intermediate is the standard replication fork, and investigations of these structures led to the identification of two distinct replication origins in mtDNA (Han and Stachow, 1994; Holt et al., 2000).

Gel electrophoresis based mtDNA analyses do not require a prior mtDNA purification step. However, they might require relatively large amounts of DNA which may pose a limitation for small tissue samples.

PCR-based techniques enable very sensitive and precise measurement of mtDNA damage and content allowing for rapid examination of large sample number starting from nanogram of template. Real-time quantitative PCR (qPCR) assay: In qPCR, a short DNA fragment, typically shorter than 200 nucleotides, is amplified in a mixture containing dNTPs, Taq DNA polymerase, and a DNA-intercalating dye. Primer design is of extreme importance for this technique. This is especially true for mtDNA studies given the presence of inactive copies of mitochondrial genes in the nuclear DNA called nuclear mitochondrial pseudogenes (NUMTs). The presence of mtDNA-like DNA segments in the nuclear genome of eukaryote cells was first suggested in 1967 (du Buy and Riley, 1967) and has subsequently been confirmed in human (Tsuzuki et al., 1983).

Quantification of the number of mtDNA molecules which correlates with the cellular energy demand of the cell and ATP production has been used to assess the state of mitochondrial function (Clay Montier et al., 2009; Fuke et al., 2011). Relative mtDNA copy numbers can be determined by qPCR using singleplex Taqman assays designed to target the mitochondrial *MT-ND1* gene and normalize it to a nuclear gene such as *B2M* (Venegas and Halberg, 2012). 18S ribosomal RNA (*18s rDNA*) is also frequently used as a control for mtDNA copy number normalizations (Filograna et al., 2019). mtDNA copy numbers can be also quantified using droplet digital PCR. In this technique, the sample is partitioned in single droplets in a water-oil emulsion to the extent that single template DNA molecules can be amplified individually. This technique provides increased precision and sensitivity compared to qPCR. Importantly, it allows the quantitation of absolute DNA copy number (Hindson et al., 2011). These assays do not provide any information about topology, deletions, or lesions in the mtDNA.

**FIGURE 3:**
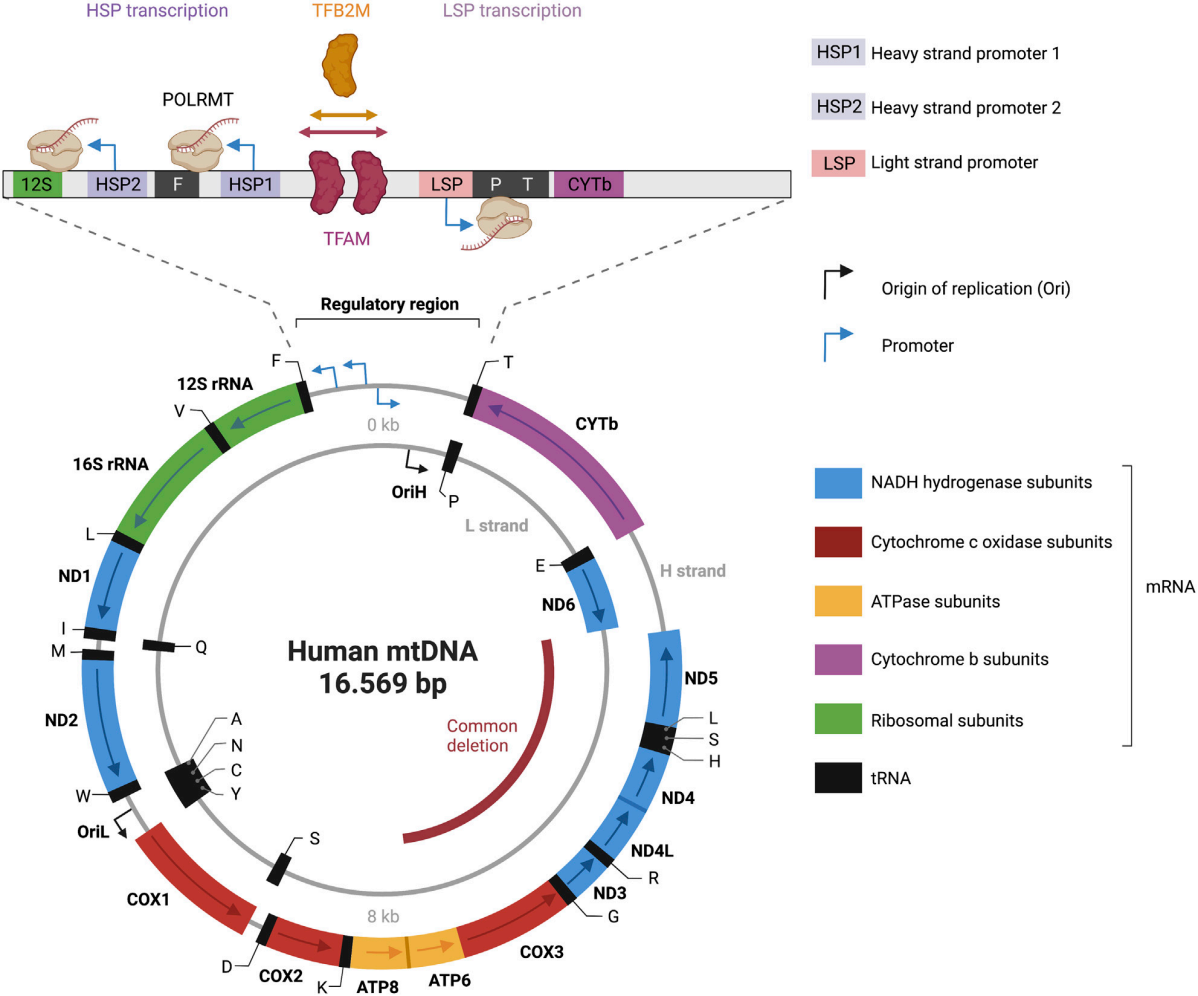
Representation of the human mitochondrial DNA transcription. Depicted is a map of the 16.569 bp circular, double-stranded mitochondrial genome. The heavy strand is depicted as outer circle and the light strand as inner circle. mRNA genes coding for NADH hydrogenase subunits are labeled in blue; mRNA genes coding for Cytochrome c oxidase subunits are labeled in red; mRNA genes coding for ATPase subunits are labeled in yellow; mRNA genes coding for cytochrome b subunits are labelled in purple; rRNA genes are labelled in green; tRNAs genes are labelled in black. Transcriptional directionality is specified for mRNAs and rRNAs. The displacement loop (D-loop) noncoding control region is depicted as expanded). D-loop encompasses three promoters: heavy-strand promoter 1 and 2 (HSP1, HSP2), and light-strand promoter (LSP). Transcription of the heavy strand initiated from HSP1 generates a short transcript terminating at the 16S rRNA. Transcription of the heavy strand initiated from HSP2, generates a polycistronic mRNA including 12 mRNA genes, 2 rRNAs and 14 tRNA genes. Light-strand transcription from the light-strand promoter (LSP) generates ND6 mRNA and 8 tRNAs. The transcription start site (TSS) of LSP, HSP1 and HSP2 has been established to be located position 407/408, 561, 643/644 respectively (Montoya et al., 1983; Chang and Clayton, 1984; Zollo et al., 2012). A comparison of the three promoter sequences revealed a mild consensus in the base pairs around the TSS with the -7A,-3C,+1 to +3AAA, and +5A being conserved positions in all three promoters (Basu et al., 2020). The transcription activator TFAM (transcription factor A, mitochondrial) and dimethyladenosine transferase 2, mitochondrial (TFB2M) are required to bind mtDNA upstream of all three promoters, recruiting a single subunit RNA polymerase (POLRMT). The figure has been generated using Biorender. com.

The multiplex *MT-ND1*/*MT-ND4* Taqman qPCR assay determines the proportion of deleted mtDNA. The assay is based on the observations that the positions of mtDNA deletions are non-random (Gokey et al., 2004; Taylor and Turnbull, 2005). Of the mtDNA deletions (reported in the MitoBreak database (Damas et al., 2014a)), loss of the MT-ND4 sequence occurs in approximately 90% of all reported break points (Damas et al., 2014b). The common deletion encompassing *MT-ND4* spans 4,977 bp (8,483–13459) (Figure 3). Therefore, amplification of this region provides a direct evaluation of deletions. *MT-ND1* gene is used for normalization as it is normally not affected by the deletion. This assay does not provide any information about mtDNA copy number or mtDNA lesions (Phillips et al., 2014).

### 2.6.2 Assays for measuring mtDNA damage

Long-PCR assay: This assay is built on the principle that DNA lesions prevent the DNA polymerase from progressing along a long DNA template, resulting in reduced amplification of the target DNA (Santos et al., 2006). This test monitors the integrity of mtDNA directly from total cellular DNA, eliminating the requirement for a mitochondria separation step. Another significant advantage of this approach is its sensitivity, which allows for the identification of about one lesion per 100,000 nucleotides. A major limitation is that it likely identifies only single- and double-strand DNA breaks, whereas lesions such as 8-oxoG, which do not significantly impede PCR DNA polymerase progression, will not be identified with high efficiency (Santos et al., 2006). Lesions which can block replicative polymerases, such as the oxidized pyrimidine 5,6 dihydroxy-5,6 dihydrothimine (Thymine glycol, Tg) (Clark and Beardsley, 1987) have also the capacity to reduce the amplification efficiency when are present in the template strand.

Quantification of nDNA or mtDNA damage can be accomplished by Real-time qPCR Analysis of Damage Frequency (RADF) (Wang et al., 2016). This method is based on the ability of a lesion in one strand to inhibit restriction enzyme TaqI digestion of double-stranded DNA. Subsequent amplification of the complementary strand after restriction cleavage gives a quantitative measure of the damage content in that site. Since Taql needs to detect double stranded TCGA sequences, DNA lesions of any of the four bases -A, T, G, or C- have the same ability to affect the cut efficiency. For this reason, this assay is informative about general DNA lesions levels while it is not informative about specific lesions.

Over the last few decades, several analytical methods for measuring DNA adducts have been developed allowing for measurement of oxidative DNA lesions by mass spectrometric techniques. Chromatographic techniques including high performance liquid chromatography with electrochemical detection (HPLC-ECD) and gas chromatography-mass spectroscopy (GC-MS) are among the analytical methods that have been developed to measure oxidative base and sugar lesions in DNA (Maynard et al., 2010; Dizdaroglu et al., 2015). In these methods, preparation of highly purified mitochondria devoid of contaminating nuclear DNA is needed. Moreover, care should be taking to avoid introducing oxidative damage to mtDNA during DNA sample purification from intact mitochondria, for instance, by including antioxidants during sample preparation. In general, although these methods can accurately measure mtDNA lesions, they need special equipment and expertise and are therefore not applicable in most clinical and research laboratories.

## 3 Mitochondrial transcription

The mitochondrial genome is transcribed in a strand-specific, bacterial-like polycistronic manner (Aloni and Attardi, 1971). The precursor polycistronic transcript is then processed to generate mature mRNAs, tRNAs and rRNAs. In a cesium chloride density gradient, the mtDNA molecule can be dissociated into two strands: a guanine-rich heavy strand and a cytosine-rich light strand (Borst, 1972) (Figure 3). The mtDNA codes for 13 messenger RNAs (mRNAs), two ribosomal RNAs (12S and 16S rRNAs), and 22 transfer RNAs (tRNAs). The mRNAs encode for NADH ubiquinone oxidoreductase subunits ND1–ND6, ND4L, cytochrome B CYTB, cytochrome oxidase subunits CO1–CO3, and ATP synthase subunits ATP6 and ATP8, which are all involved in the OXPHOS.

### 3.1 Mitochondrial transcription initiation

Sequencing results in the early 1980s revealed that the mitochondrial genome is incredibly compact. MtDNA is mostly coding (>90%) and both strands harbor different amounts of genetic information (Anderson et al., 1981). The only noncoding region in mtDNA is a 1.1-kb region called displacement loop (D-loop), that features most of the known transcription and replication regulatory elements (Nicholls and Minczuk, 2014). Three promoters are located within a 250-bp section of the D-loop: two heavy-strand promoters (HSP1 and HSP2), and a light-strand promoter (LSP) (Figure 3). The closely spaced HSP1 and HSP2 promoters in the heavy strand drive the expression of 12 mRNAs, two rRNAs, and 14 tRNAs. The LSP drives the expression of one mRNA and eight tRNAs. HSP and LSP drive transcriptions in opposite directions.

DNA-directed RNA polymerase (POLRMT) catalyzes the transcription of a polycistronic RNA transcript, which is then processed to yield mature RNAs. POLRMT shows a significant structural and sequence similarity to the RNA polymerases found in T3 and T7 bacteriophages (Cermakian et al., 1997; Tiranti et al., 1997; Ringel et al., 2011).

The C-terminal domain (CTD) structure of POLRMT constitute the catalytic core and resembles the classic “right hand” shape composed of the thumb, palm, and fingers domains (Jeruzalmi and Steitz, 1998; Cheetham et al., 1999; Ringel et al., 2011). The palm and fingers domains contain the active site responsible for catalyzing nucleotide incorporation, while the thumb domain is essential for DNA binding (Sousa et al., 1993). The N-terminal domain (NTD) contains two promoter-recognizing structural elements, the AT-rich recognition loop and the intercalating hairpin (ICH); the third element, the specificity loop, is in the CTD. The NTD also contains two pentatricopeptide repeat (PPR) typical of RNA-associated proteins and necessary for site-specific interactions (Ringel et al., 2011; Barkan et al., 2012).

The promoter-binding elements in POLRMT have diverged from T7 RNAP causing reduced promoter sequence recognition capacity and increasing reliance on transcription factors. Dimethyladenosine transferase 2, mitochondrial (TFB2M), is required for transcription from all three promoters (Morozov et al., 2014). There are significant differences in the transcription initiation mechanism at the three promoters that likely arise from the differences in promoter sequences and their requirement for TFAM. TFAM molecules can attach to the region between the HSP1 and LSP and regulate the two promoters (Uchida et al., 2017). *In vitro* studies indicate that LSP and HSP1 require TFAM for optimal transcription (Litonin et al., 2010; Ramachandran et al., 2017), whereas TFAM inhibits transcription from HSP2 (Lodeiro et al., 2012; Zollo et al., 2012).

Once POLRMT and the transcription factors bind at the promoter regions they form a transcriptional complex and catalyze the RNA synthesis. TFAM can specifically bind to LSP (Fisher and Clayton, 1985; Uchida et al., 2017), and can form a complex with POLRMT on the DNA (Morozov et al., 2014). Mechanistically it is proposed that TFAM recruits POLRMT to LSP promoter forming a pre-initiation complex. POLRMT and TFB2M alone can bind with high efficiency and catalyze promoter proximal transcription (Basu et al., 2020). However, it is not clear how this model differs for the HSP1 and HSP2 (Basu et al., 2020). The initiation complex structure with POLRMT, TFAM, and TFB2M shows that the three proteins cover the promoter site (Hillen et al., 2017a).

### 3.2 Mitochondrial transcription elongation and termination

For the elongation stage, POLRMT requires transcription elongation factor (TEFM) (Minczuk et al., 2011; Hillen et al., 2017b) which promotes processivity stimulating the longer transcript formation *in vitro* (Posse et al., 2015) as its depletion leads to a reduction in promoter–distal transcription elongation products (Minczuk et al., 2011). Transcriptional termination has been proposed to be dependent on MTERF1 which binds within the tRNA encoding leucine downstream the rRNAs genes downstream HSP promoters (Kruse et al., 1989; Asin-Cayuela et al., 2005; Guja and Garcia-Diaz, 2012).

### 3.3 Transcription-associated BER: From nuclear to mitochondrial gene expression regulation

**FIGURE 4:**
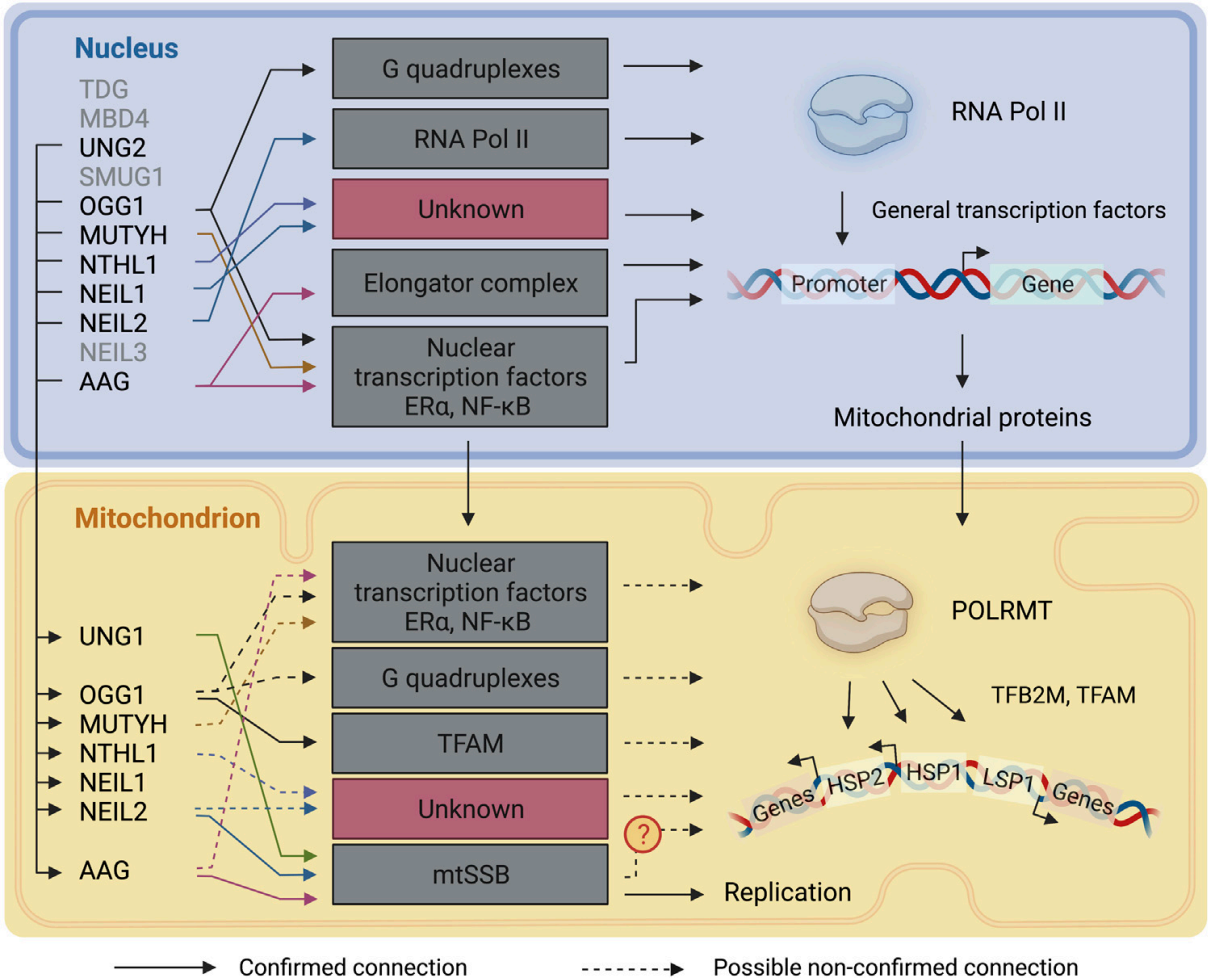
DNA glycosylases involvement in transcriptional regulation. A representation of the connections between DNA glycosylases and the transcription mechanisms in the nucleus and mitochondria. Continuous lines represent connections that have been described. Dashed lines represent connections that are likely to take place but have not been directly described. TDG: Thymine-DNA glycosylase; MBD4: Methyl-CpG-domain protein 4; UNG1/2: Uracil-DNA glycosylase; SMUG1: Single-strand selective monofunctional uracil DNA glycosylase 1; OGG1:8-Oxoguanine-DNA glycosylase 1; MUTYH: Adenine-DNA glycosylase; NTHL1: Endonuclease III-like protein 1; NEIL1: Nei endonuclease VIII-like 1; NEIL2: Nei endonuclease VIII-like 2; NEIL3: Nei endonuclease VIII-like 3; AAG: Alkyladenine-DNA glycosylase. RNA Pol II: RNA polymerase II: POLRMT: DNA-dependent RNA polymerase; TFAM: mitochondrial transcription factor A; TFB2M: Dimethyladenosine transferase 2, mitochondrial; HSP: heavy-strand promoter; LSP: light-strand promoter. The figure has been generated using Biorender. com.

Unrepaired DNA damage can influence cells via mutagenesis and beyond. Base damage, for example, can influence the affinities of DNA binding proteins, such as transcription factors, and interfere with transcription fidelity, resulting in modified transcripts when damage is bypassed by the transcription machinery. While complex DNA lesions can induce cytotoxic effects by blocking polymerases (Bjelland and Seeberg, 2003), base damage, albeit with some important exceptions, does not generally cause DNA helix distortions and in most instances does not result in RNA polymerase stalling during transcription elongation in the nucleus (Lans et al., 2019). Base lesions can however cause polymerase pausing or alter fidelity (Charlet-Berguerand et al., 2006; Agapov et al., 2022). It is safe to assume that, similarly, base damage does not block POLRMT during mitochondrial transcription elongation but might cause pausing or fidelity alterations. On the other hand, unrepaired base lesions which are recognized and excised during transcription, and interference between DNA repair and transcription, may lead to the exposure of transcription-blocking DNA repair intermediates such as AP sites or single strand breaks (SSB). Although little is known about the impact of a single DNA base damage on transcription efficiency, current research supports the idea of a nuclear transcription associated-BER, given preferential base repair on actively transcribed regions. Here we discuss the plausibility of a transcription associated-BER in mitochondria (Figure 4).

Canugovi et al. showed that TFAM preferentially binds DNA containing 8-oxoG substrate over normal DNA (Canugovi et al., 2010), but did not show any preference for uracil, AP sites or gap-containing substrates. Furthermore, when tested for its ability to modulate *in vitro* BER enzymatic activities, TFAM inhibited OGG1, UDG, APE1, pol γ and, additionally, OGG1 in a cell-based assay (Canugovi et al., 2010). The same work showed that TFAM’s interaction with p53 modifies TFAM DNA binding and has the capacity to promote BER in mtDNA. In other words, TFAM could be an important regulator of BER supporting the model where association of TFAM with DNA can regulate BER proteins access to the DNA. As the major functions of TFAM are to compact mtDNA and regulate transcriptional initiation, this work highlights the possibility of a link between BER and transcription in mtDNA.

#### 3.3.1 8-Oxoguanine-DNA glycosylase 1 (OGG1) and adenine-DNA glycosylase (MYH)

8-oxoguanine-DNA glycosylase 1 (OGG1) repairs oxidized pyrimidines, with 8-oxoG being the classical substrate. Some reports suggest that OGG1 is also able to excise ring-opened formamidopyrimidines FapyG and FapyA from DNA (Krishnamurthy et al., 2008; Ba and Boldogh, 2018). The mitochondrial isoform, OGG1-β, differs from the nuclear isoform OGG1-α, as it lacks the nuclear localization signal near the C- terminus encoded by exon 7. Exon 8 replaces exon 7 in the OGG1-β mRNA (Nishioka et al., 1999). The importance of a mitochondrial OGG1 is illustrated by the fact that mtDNA accumulates high levels of 8-oxoG (Mecocci et al., 1993; Hudson et al., 1998). Furthermore, 8-oxoG levels in mtDNA isolated from liver, heart and brain were 6-, 16- and 23-fold higher than nDNA, suggesting that tissues with high oxidative load accumulate more oxidative DNA damage in mtDNA than in nuclear DNA (Hamilton et al., 2001). 8-oxoG accumulation in mtDNA is increased in *Ogg1* ^*−/−*^ mice compared to WT thus suggesting that repair depended on OGG1 (de Souza-Pinto et al., 2001). Correspondingly, mitochondrial targeted human OGG1 was shown to enhance mitochondrial DNA repair, increase mitochondrial function, and protect different cell types from oxidant mediated death (Rachek et al., 2002; Yuzefovych et al., 2012; Yuzefovych et al., 2013).

In the nucleus, recruitment of OGG1 on 8-oxoG lesions in DNA can alter gene expression through three different mechanisms (reviewed in (Wang et al., 2018; Bordin et al., 2021)):

(1) The first mechanism involves secondary DNA structures. Depending on certain sequence motifs and interactions with other proteins, DNA can adopt a variety of alternative conformations in addition to the right-handed double helix shape. G-quadruplexes (G4) are among these secondary structures and are formed when G-quartet - four guanine molecules form a square planar arrangement in which each guanine is hydrogen bonded to the two adjacent guanines are stacked. Recent evidence suggests involvement of G4 structures in transcription, replication, genome stability, and epigenetic regulation. 8-oxoG accumulation in G4 forming sequences can influence transcription depending in which strand the lesion occurs. In the coding strand, after OGG1 recognition, APE1 recruits transcription factors to the site, enhancing transcription (Fleming et al., 2017a). In the template strand, the 8-oxoG processing through OGG1 and APE1 leads to transcription inhibition (Fleming et al., 2017b; Zhu et al., 2018). APE1 modulates G4-mediated expression of genes as its binding to AP sites promotes G4 folding (Roychoudhury et al., 2020). G-quadruplexes are not exclusive structures of nuclear DNA but can occur also in mtDNA (Dong et al., 2014) and thus influence the regulation of mitochondrial gene expression (Falabella et al., 2019). Thus, it is possible that OGG1 can interact with these structures in mtDNA as well, but data on the subject is lacking.
(2) A second mechanism that suggests OGG1 influences transcription is the recognition of 8-oxoG at the promoters of NF-κB target genes. OGG1 binding facilitates DNA bending at the site, enhancing NF-κB occupancy and recruitment of specific transcription factors (Ba et al., 2014; Ba and Boldogh, 2018). Mitochondrial localization was recently described for the proteins p50/NF-kB1 and RelA (p65), two subunits of the NF-kB family of transcription factors (Barshad et al., 2018). Furthermore, in mouse keratinocytes, STAT3 bound mtDNA and co-immunoprecipitated with TFAM. STAT3 knockout increased the transcription of several mitochondrial genes, pointing to a potential inhibitory role of STAT3 in mtDNA transcription (Macias et al., 2014).
(3) In a third mechanism, 8-oxoG regulates chromatin relaxation and remodeling in estrogen- and retinoic acid-induced genes (Perillo et al., 2008; Zuchegna et al., 2014). Estrogen receptors class α and β (ERα and ERβ, respectively) are transcriptional activators which are primed by estradiol (estrogen hormone family) binding. In the nucleus, ER targeted demethylation of histone 3 lysine 9 (H3K9) via local activation of LSD1 (Lysine demethylase 1A) causes local formation of H_2_O_2_ followed by a rapid highly controlled increment of 8-oxoG in discrete foci in proximal promoters and enhancers. OGG1 together with topoisomerase IIβ initiates the BER pathway, that generates the required nicks used by topoisomerases to relax and open the chromatin for estrogen mediated transcription (Perillo et al., 2008). ERs have been shown to localize to mitochondria and bind putative estrogen-response elements within mtDNA, and there is evidence that ERs may directly regulate the expression of both mitochondrial- and nuclear encoded OXPHOS subunit genes (Klinge, 2020). ERs have been shown to indirectly up-regulate mtDNA-encoded OXPHOS subunits through the NRF/TFAM pathway (Pronsato et al., 2020). Although indirectly these findings suggest that OGG1 could regulate effectors of mitochondrial transcription through already described nuclear mechanisms more studies are necessary to confirm these putative mechanisms.

More direct evidence of OGG1 involvement in mitochondria showed that in OGG1^Tg^ mice constitutively overexpressing human OGG1 in the mitochondria, the expression of *Tfam* as well as several mitochondrial transcripts were significantly upregulated, indicating increased transcription of mitochondrially-encoded genes in these animals compared to the WT mice (Komakula et al., 2018). mtDNA 8-oxoG immunoreactivities colocalize with TFAM upon exposure to menadione in MEFs lacking mitochondrial Ogg1 (Oka et al., 2008).

MYH recognizes A:G, A:C and A:8-oxoG mispairs. While the effect of MYH alone on transcription has not been described, transcriptome analysis from *Ogg1* ^*−/−*^ *Myh* ^*−/−*^ mice hippocampi showed upregulation of ERα target genes involved in anxiety and cognition regulation, thus suggesting that the two DNA glycosylases might act in concert to regulate ERα signaling (Bjorge et al., 2015). It is not known if a similar mechanism is present in mitochondria.

#### 3.3.2 Endonuclease III-like protein 1 (NTHL1) and endonuclease 8-like 1–2 (NEIL1-2)

Endonuclease III-like protein 1 (NTHL1) has glycosylase activity on oxidized pyrimidines 5,6 dihydroxy-5,6dihydrothimine (Thymine glycol, Tg), 2,6-diamino-4-oxo-5-formamidopyrimidine (FapyG), 4,6-diamino-5-formamidopyrimidine (FapyA), 5-hydroxyuracil (5-ohU), 5-hydroxycytosine (5-ohC) as well as 8-oxoG (Michaels et al., 1992; Aspinwall et al., 1997; Hilbert et al., 1997; Ikeda et al., 1998). The mitochondrial NTHL1 activity is higher than their nuclear isoforms (Karahalil et al., 2002). *C. elegans,* is equipped with only two DNA glycosylases: endonuclease III-1 (NTH-1) ortholog of human NTHL1 and uracil DNA glycosylase-1 (UNG-1). NTH-1 has been shown to be a key mediator of age-dependent genomic instability and compromised NTH-1 activity promotes neuroprotection in a Parkinson disease model in nematodes as a direct effect of defective BER in mitochondria. Importantly, loss of NTH-1 activity altered mitochondrial transcription output inducing general overexpression of mitochondrial genes compared to control in an age-dependent manner (SenGupta et al., 2021). This evidence suggests that NTH-1 can regulate mitochondrial transcription in *C. elegans* and hints the existing of a similar mechanism in humans. However, NTH-1 is the only DNA glycosylase involved in the repair of oxidative DNA damage in *C. elegans* (Elsakrmy et al., 2020), while other DNA glycosylases with comparable substrate selectivity to NTHL1 are present in humans (comparison between eukaryotic organisms reviewed in (Hindi et al., 2021)), suggesting a more complex control, dependent on several DNA glycosylase.

Endonuclease 8-like 1 and 2 (NEIL1 and NEIL2) have similar substrate specificity. NEIL1 was originally shown to recognize Tg, 5,6-dihydrothymine (DHT), 5,6-dihydrothymine (DHU), 5-ohC, FapyG and FapyA (Hazra et al., 2002a; Bandaru et al., 2002; Jaruga et al., 2004). NEIL2 is active on 5-ohU, 5-ohC as well as Tg, DHT and DHU (Hazra et al., 2002b; Wallace et al., 2003).

NEIL1 and NEIL2 have activity on bubble structured or ssDNA *in vitro*, suggesting preferential repair activity in actively transcribed DNA (Dou et al., 2003). NEIL2 in complex with the transcription machinery repairs lesions preferentially in the transcribed genes and not on transcriptionally silent genes in mammalian cells (Englander and Ma, 2006; Banerjee et al., 2011). Consistently, in shRNA-NEIL2 stable cell lines, transcribed genes showed significant increase in DNA lesion accumulation in comparison to non-transcribed genes (Banerjee et al., 2011).

NEIL2 was shown to localize and to be active in mitochondria (Mandal et al., 2012) and chromatin immunoprecipitation analysis showed NEIL2 binding to the mitochondrial genes MT-CO2 and MT-CO3 ^216^. Neil2-morphant whole embryos of *Xenopus laevis* showed upregulation of mitochondrial (mt) gene expression (namely *mt-Nd1,4* and *5*, *mt-co3*) as response to mitochondrial dysfunction (Han et al., 2019). Further, NEIL1 interacts with mitochondrial single-stranded binding protein (mtSSB1), an essential protein for mtDNA replication (Jiang et al., 2021). MtSSB1 defects cause transcriptional alterations including hypoxia response in *C. elegans* (Sugimoto et al., 2008) highlighting that this protein might be involved in mitochondrial transcription. MtSSB1 interacts with NEIL1 to inhibit its AP-lyase activity (Sharma et al., 2018).

#### 3.3.3 Alkyladenine DNA glycosylase (AAG)

Alkyladenine DNA glycosylase (AAG), also called N-methylpurine DNA glycosylase (MPG) recognizes and excises alkylated bases 7-methylguanine (7-meG), 3-methyladenine (3-meA) and 3-methylguanine (3-meG). These adducts can be generated using the alkylating agent methyl methane sulfonate (MMS) (Chakravarti et al., 1991; Samson et al., 1991; Alseth et al., 2006). AAG is also able to remove hypoxanthine (Hx) from DNA. In the nucleus, AAG interacts with several transcription factors. One interactor is estrogen receptor alpha (ERα). AAG modulates ERα capacity to bind Estrogen response elements (ERE) containing genes, resulting in the repression of transcription, (Likhite et al., 2004). ERα influences AAG capacity to repair Hx containing DNA. Another AAG interactor is methylated DNA-binding domain 1 (MBD1), a repressor of transcription able to recognize methylated regions. Once an AAG-MBD1 complex is established they repress methylated promoters and facilitate AAG mediated repair of MMS induced lesions (Watanabe et al., 2003). AAG has been shown to interact with RNA pol II through the interaction with the Elongator complex. This interaction allows the recruitment of BER components to the chromatin to complete the AAG-initiated repair in a transcription coordinated manner (Montaldo et al., 2019). This suggests that AAG influences nuclear transcription to repair aberrantly methylated bases.

In the mitochondria, AAG interacts with mtSSB which inhibits AAG repair activity in single-stranded, but not in double-stranded DNA (van Loon and Samson, 2013). The consequences of AAG-mtSSB interaction have not been explored further and it is not clear whether AAG is able to interact with the mitochondrial replication or transcription machinery. Loss of AAG does not seem to have any effect on mitochondrial dysfunction caused by alkylative DNA damage (Alhumaydhi et al., 2020). Further studies are required to address the impact of this glycosylase on mitochondrial transcription.

#### 3.3.4 Uracil-DNA glycosylase (UNG)

Uracil-DNA glycosylase (UNG) removes uracil from U:A pairs that arise from incorporation of dUMP in the place of dTMP during DNA replication (nuclear and mitochondrial), premutagenic U:G mismatches that are formed from spontaneous or enzymatic deamination of cytosine, and uracil in single-stranded DNA (Sekiguchi et al., 1976; Olsen et al., 1989; Liu et al., 2002; Krokan and Bjoras, 2013).

The human *UNG* gene encodes both nuclear (UNG2) and mitochondrial (UNG1) isoforms of the enzyme by a mechanism involving two different promoters and alternative splicing. The mRNAs for UNG2 and UNG1 encode 44 and 35 unique N-terminal residues that are required for mitochondrial and nuclear translocation, respectively, whereas the downstream 269 residues encompassing the catalytic region of the enzyme are common. Therefore, the two isoforms have the same catalytic activity (Slupphaug et al., 1993; Nilsen et al., 1997; Otterlei et al., 1998). Mitochondrial UNG1 activity has been reported (Karahalil et al., 2002). Interestingly, mtSSB completely inhibits uracil-excision by UNG from single-stranded DNA (Wollen Steen et al., 2012).

Patients carrying inactivating mutations in the *UNG* gene show skewed somatic hypermutation and reduced class-switch recombination mechanism (Imai et al., 2003). However, whether these patients show symptoms of mitochondrial dysfunction has not been reported.

#### 3.3.5 Apurinic/apyrimidinic endonuclease (APE1)

APE1 acts downstream of DNA glycosylases and incises at the 5′-end of AP sites. APE1 AP-site cleavage is inhibited by mtSSB (Wollen Steen et al., 2012). Photodynamic therapy (PDT), a promising cancer treatment strategy, generates ROS using a laser in the presence of photosensitizers. Mitochondrial dysfunction is thought to be one of the major causes of cell death upon PDT. Protein–protein interaction experiments showed that, upon PDT, APE1 localizes to the mitochondria where it interacts with TFAM and TFB2M and regulates TFAM transcriptional activity (Li et al., 2012). APE1 was independently identified as a reductive function as a redox effector factor (Ref-1) for several transcription factors including AP-1 (c-Jun/Fos heterodimer), hypoxia-inducible factor HIF1-α, and p53 (Xanthoudakis and Curran, 1992; Tell et al., 2005; Bhakat et al., 2009). Consequently, APE1 could operate as a redox factor enzyme in regulating transcription the mitochondria.

## 4 Conclusion

The importance of mitochondrial functions is highlighted by their multiple cellular roles, complex relationship with the nucleus, and the wide spectrum of illnesses linked with their malfunction. Beyond its crucial function in the preservation of nuclear and mtDNA, BER has been demonstrated to contribute to the regulation of nuclear transcription (Figure 4). While some data suggests that this might also be true in mitochondria, the importance of BER in mitochondrial transcription is still elusive (Figure 4). Two essential mitochondrial proteins involved in mtDNA metabolism appear to interact with the BER pathway. TFAM, an important factor for packaging mtDNA and for transcriptional initiation, influences BER enzymatic activity in a replication- and transcription-dependent manner. A second factor, mtSSB, important for mtDNA replication and perhaps also transcription, interacts with several DNA glycosylases. Defect in mtssb1-causes transcriptional alterations in *C. elegans* (Sugimoto et al., 2008), hinting at the possibility of a tight regulation of transcription, replication, and DNA repair in mitochondria. Thus, more research is warranted to investigate the possible role of BER in mitochondrial transcription regulation, which could aid to develop therapeutic avenues for mitochondrial diseases caused by loss of mtDNA and transcription integrity.
